# Importance of the Habenula for Avoidance Learning Including Contextual Cues in the Human Brain: A Preliminary fMRI Study

**DOI:** 10.3389/fnhum.2020.00165

**Published:** 2020-05-12

**Authors:** Atsuo Yoshino, Yasumasa Okamoto, Yuki Sumiya, Go Okada, Masahiro Takamura, Naho Ichikawa, Takashi Nakano, Chiyo Shibasaki, Hidenori Aizawa, Yosuke Yamawaki, Kyoko Kawakami, Satoshi Yokoyama, Junichiro Yoshimoto, Shigeto Yamawaki

**Affiliations:** ^1^Department of Psychiatry and Neurosciences, Hiroshima University, Hiroshima, Japan; ^2^Division of Information Science, Graduate School of Science and Technology, Nara Institute of Science and Technology, Nara, Japan; ^3^Department of Neurobiology, Hiroshima University, Hiroshima, Japan; ^4^Department of Cellular and Molecular Pharmacology, Hiroshima University, Hiroshima, Japan; ^5^Center for Brain, Mind and KANSEI Sciences Research, Hiroshima University, Hiroshima, Japan

**Keywords:** habenula, fMRI, depression, chronic pain, hippocampus

## Abstract

Human habenula studies are gradually advancing, primarily through the use of functional magnetic resonance imaging (fMRI) analysis of passive (Pavlovian) conditioning tasks as well as probabilistic reinforcement learning tasks. However, no studies have particularly targeted aversive prediction errors, despite the essential importance for the habenula in the field. Complicated learned strategies including contextual contents are involved in making aversive prediction errors during the learning process. Therefore, we examined habenula activation during a contextual learning task. We performed fMRI on a group of 19 healthy controls. We assessed the manually traced habenula during negative outcomes during the contextual learning task. The Beck Depression Inventory-Second Edition (BDI-II), the State-Trait-Anxiety Inventory (STAI), and the Temperament and Character Inventory (TCI) were also administered. The left and right habenula were activated during aversive outcomes and the activation was associated with aversive prediction errors. There was also a positive correlation between TCI reward dependence scores and habenula activation. Furthermore, dynamic causal modeling (DCM) analyses demonstrated the left and right habenula to the left and right hippocampus connections during the presentation of contextual stimuli. These findings serve to highlight the neural mechanisms that may be relevant to understanding the broader relationship between the habenula and learning processes.

## Introduction

The habenula is an epithalamic nucleus situated between the dorsal posterior thalamus and the third ventricle near the posterior commissure (Hikosaka, 2010). It is involved in responding to aversive prediction error coding, reception or prediction of aversive stimuli, and avoidance learning processing (Hikosaka, 2010; Hennigan et al., 2015). The responses in the habenula facilitate flexible responding and choice-making under various conditions (Hikosaka, 2010) through the inputs from regions such as the lateral hypothalamus, globus pallidus, and medial prefrontal cortex, and outputs to structures such as the dopaminergic ventral tegmental area (VTA), substantia nigra (SN), and serotonergic raphe nuclei, which inhibit dopamine and serotonin neurons (Matsumoto and Hikosaka, 2009; Proulx et al., 2014). Thus, the habenula enables experienced aversive sensory and internal states in terms of negative motivation and emotions to trigger a switch in behavioral actions (Hikosaka, 2010), particularly avoidance *via* inhibition of the reward signaling midbrain dopamine system. Given the major role of the habenula in motivation, emotions, and behaviors, the structure has been associated with the etiology of chronic pain and a variety of psychiatric disorders such as depression and anxiety disorders (Boulos et al., 2017; Fakhoury, 2017). However, the relationship between human habenula functioning and these various emotional and behavioral outcomes remains unclear.

Various functional magnetic resonance imaging (fMRI) studies have examined roles in the human habenula by using a high-resolution fMRI approach. For example, Lawson et al.’s (2017) work observed activation during the prediction of aversive stimuli. They conducted a passive (Pavlovian) conditioning task using both reward (monetary gain) and aversive (monetary loss and electric shock) stimuli (Lawson et al., 2017). They showed that the habenula was highly activated coincident with a conditioned stimulus (CS) associated with electric shock in healthy participants. The prediction of aversive stimuli requires preliminary prior phases for learning, but it is difficult to clarify how the prediction is related to learning and behavior. Furman and Gotlib (2016) have examined human habenula activation using a probabilistic guessing task for monetary rewards and penalties. They observed that there was greater activation of the left and right habenula during experience of monetary loss (penalties) than win outcomes (rewards). These studies have shown increased habenula activation in healthy participants during aversive stimuli and anticipation of noxious stimuli. However, they were unable to reveal a possible relationship between the habenula and learning processing for adjusting adaptive behavioral actions to reinforcers, although such a relationship should clarify another important role of the habenula.

The habenula obtains comprehensive current internal or external state information from many brain regions and then works to guide behavioral responses to predicted error signals, as learning processing (Matsumoto and Hikosaka, 2009; Proulx et al., 2014). Animal studies have suggested that the habenula would be particularly important for learned strategies such as those involved in aversive prediction errors. For example, Kawai et al. (2015) have examined the behavioral performance and habenula functioning in monkeys using a reversal-learning task, and have confirmed habenula activation during aversive prediction errors. A reversal-learning task elicits aversive prediction errors when a target associated with reward throughout multiple trials suddenly stops being associated with reward. Aversive prediction errors are interpreted as a signal indicating that an expected reward might be less than predicted, or that aversive stimuli might be experienced instead of the expected reward. Such information contributes to subsequent behavioral adjustments such as changes in future behavioral choices. In a human study about such a learning approach, Liu et al. (2017) investigated habenula activation during a probabilistic reinforcement learning task. The left habenula was highly activated during punishment conditions in healthy participants. However, this previous experimental task just constituted simple cue conditioning, and aversive prediction errors were very rare under such conditions.

Growing evidence from rat studies suggest that the habenula plays a more important role in flexible switching from the conventional strategy when contextual contingencies change than for processing of simple aversive signals (Baker et al., 2015; Mizumori and Baker, 2017). Contextual information in learning has been regularly and indirectly displayed on a background as an unexpected predictor of the unconditioned stimulus (US) throughout the task, and such information has been shown potentially or subconsciously for participants. Therefore, it has been thought to generate aversive prediction errors by providing information without intention and awareness of what has been learned (Goujon et al., 2015). Cue conditioning has been generally defined as the pairing of a discrete cue such as a painful stimulus with the US, whereas contextual conditioning has been implicitly recognized as the pairing of internal or external state information such as a spatial perception with the US. A previous animal study has shown that the habenula in rats would be implicated in the processing of contextual learning and memory (Chan et al., 2017). Human fMRI studies also demonstrated that fear learning processing was acquired by presenting aversive stimuli and contextual visual information simultaneously (Pohlack et al., 2012; Steiger et al., 2015). However, to date, there has been no human fMRI studies on habenula activation that has incorporated contextual factors. Extrapolating from the above studies involving contextual learning tasks that elicit aversive prediction errors may lead to clarification of neural mechanisms in the human habenula. Therefore, we examined aversive prediction errors related functionality of the habenula using a high-resolution fMRI analysis of a learning task that involves an effect of contextual information on behavioral choices as a preliminary study. Based on previous studies, we hypothesized that the habenula would activate when negative outcomes and aversive prediction errors become apparent, and that functional activation of the habenula would be associated with progress in learning.

Furthermore, previous animal studies have suggested that the hippocampus would functionally link to the habenula, particularly during contextual conditioning, and that its region would play an important role in habenula neural activity (Loonen and Ivanova, 2016; Mizumori and Baker, 2017). Mizumori and Baker (2017) propose a habenula network that includes this region and the hippocampus, with this network involved in contextual conditioning. Lesions of the habenula resulted in impaired hippocampus-mediated learning and memory in rat studies (Thornton and Davies, 1991; Chan et al., 2017). Moreover, the involvement of the hippocampus has been strongly suggested to play an essential role in implicit learning mechanisms as the neural bases of contextual conditioning (Goujon et al., 2015). Therefore, we also assessed functional connectivity between the habenula and hippocampus during the presentation of contextual stimuli using dynamic causal modeling (DCM) analysis.

Previous animal studies have shown that individual differences in temperament in rats were related to habenula activation (Shumake et al., 2003, 2005). They estimated three temperaments that were associated with behavioral phenotypes in punishment learning tasks in congenitally helpless rats (Shumake et al., 2005). They used reward dependence, novelty-seeking, and behavioral inhibition outlined in Cloninger’s theory as the temperaments (Cloninger, 1987). Reward dependence is characteristic of acute responding to reward signals leading to approach behavior; novelty seeking is defined as the tendency to act in response to novel stimuli resulting in exploratory behavior, and behavioral inhibition is defined as acute responding to aversive stimuli leading to passive avoidance. The results indicated that congenitally helpless rats with increased activation of the habenula were characterized by low reward dependence, high novelty seeking, and high behavioral inhibition. Moreover, they suggested that these features might be predisposing factors in individual activation of the habenula that is associated with aversive learning tasks (Shumake et al., 2003, 2005). However, this phenomenon has not been reported in human studies. Therefore, we examined the relationship between the activation of the habenula in punishment learning and the above-mentioned temperaments by using the Temperament and Character Inventory (TCI), which is an inventory that is developed based on Cloninger’s theory. We hypothesized that higher activation of the habenula during the process of aversive learning would be related to individual characteristics including low reward dependence, high novelty seeking, and high behavioral inhibition.

## Materials and Methods

### Participants

Nineteen healthy adults (13 females and six males, M age = 31.3 years, range = 21–58 years, all right-handed) participated in the present study. The participants’ self-reports indicated that they had no physical problems or a history of psychiatric disorders. We set a minimum number of participants according to previous habenula fMRI studies that used pain stimuli (Shelton et al., 2012; Hennigan et al., 2015). The previous studies have shown that the measured effect size of habenula activation by pain stimuli was 0.66 (Shelton et al., 2012) and 0.78 (Hennigan et al., 2015), and the mean effect size was 0.72. For a statistical test to detect this association with power = 0.80 and *α* = 0.05, we determined that the statistical test would need a minimum of 17 participants. All subjects gave their written informed consent before participation, according to a protocol approved by the ethics committee of Hiroshima University. We assert that all procedures contributing to this work comply with the ethical standards of the ethics committee of Hiroshima University and with the Helsinki Declaration of 1975, as revised in 2008.

**Figure 1:**
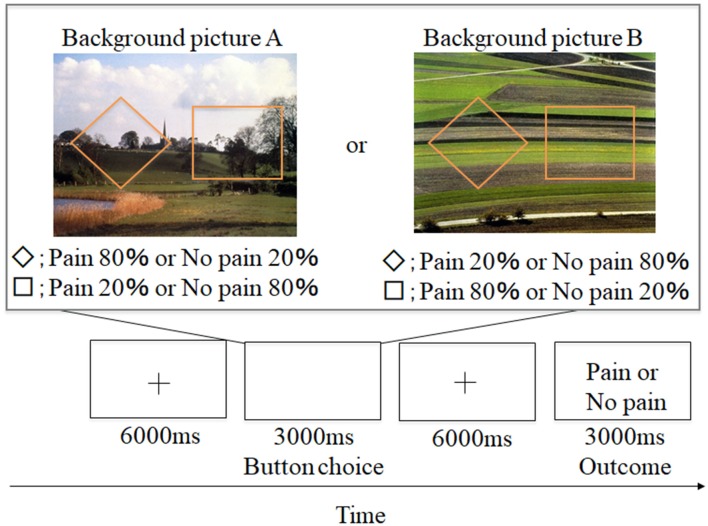
Example of aversive-based trials in the contextual learning task. A total of 60 trials was conducted. + grid point.

### Clinical Assessments

#### Psychometric Evaluation

The Beck Depression Inventory-Second Edition (BDI-II) was used to measure depressive symptoms (Beck et al., 1996). The State-Trait-Anxiety Inventory (STAI) was also administered (Spielberger, 1983). TCI consists of 125 questions with four possible answers, and the reliability and validity of the Japanese version of the TCI have been confirmed in different Japanese populations (Kijima et al., 1996). The TCI evaluates personality in terms of a 7-factor psychobiological model comprising four temperament dimensions (novelty seeking, harm avoidance, reward dependence, and persistence) and three character dimensions (self-directedness, cooperativeness, and self-transcendence). All subjects completed the BDI-II (6.0 ± 7.9), the STAI (STAI-state = 37.7 ± 9.3, STAI-Trait = 42.4 ± 11.8), and the TCI before fMRI recording.

#### Contextual Learning Task

Participants conducted an instrumental punishment-based contextual learning task. A schematic representation of the experimental design is shown in Figure 1. Two shapes (a square and rhombus) were presented for 3,000 ms. Participants were instructed to select either shape during the presentation. Afterward, a grid point (+) was shown for 6,000 ms, and an outcome of a painful stimulus or no stimulus was presented while showing word information (e.g., “pain” or “no pain” in Japanese). These shapes appeared against one of two background pictures. Participants were told that the shapes and pictures would not be switched out for new shapes or pictures. The two background pictures used (picture A and picture B) were selected from the International Affective Picture System (IAPS; Lang et al., 1997), and we adopted them because of the almost equivalent valence and arousal ratings [No. 5711; valence = 6.62, arousal = 3.03 (picture A) and No. 5720; valence = 6.31, arousal = 2.79 (picture B)]. The background picture presentations and shape location were randomized, and the number of each background presentation was adjusted to be equal (each trial number of picture A and picture B was 30). In the experiment, the probability of painful stimulus appearance differed by selected shape (a square or rhombus). Furthermore, the probability also varied for each background picture. For example, for background picture A, when participants selected a rhombus, they received a painful stimulus with an 80% probability, and when they selected a square, they received a painful stimulus with a 20% probability. For background picture B, when participants selected a square, they received a painful stimulus with a 20% probability, and when they selected a rhombus, they received a painful stimulus with an 80% probability. This information was not made explicit to participants. However, we informed the participants that the probability itself was stable but some factors could reverse this situation. This instruction was conducted to encourage them to figure out the meaning of this task through trial and error. Participants were required to associate the specific shapes with painful stimuli through trial and error selections and were instructed to avoid painful stimuli as much as possible.

**Figure 2:**
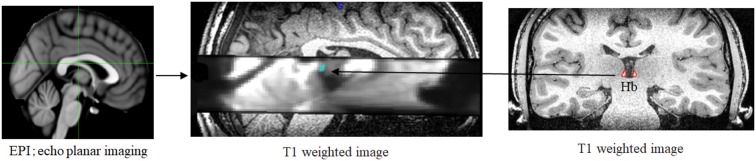
Example of individual data. The T1 structural images to enable localization of the habenula and echo-planar imaging (EPI) were co-registered to their whole-brain anatomical scans, respectively.

An intraepidermal stimulation method was used to induce minor pain at the superficial skin level (Inui and Kakigi, 2012), and we previously used this method to enact painful stimuli (Yoshino et al., 2010, 2012, 2013). We used a stainless steel concentric bipolar needle electrode (Nihon Kohden, Tokyo, Japan) for intraepidermal stimulation. The anode was an outer ring 1.2 mm in diameter, and the cathode was an inner needle that protruded 0.1 mm from the outer ring. The electrical stimuli used were 50 Hz current constant double pulses of 0.5 ms in duration. We stimulated the left forearm of each participant. Each of the subjects rated pain stimulus intensity on a verbal scale from 0 (no pain) to 10 (extremely intense pain) outside the MRI room before imaging was conducted, and a current intensity that corresponded to a rating of 5 (moderate pain) was used during the later imaging phase.

#### fMRI Acquisition

The fMRI procedure was performed using a 3.0T SIEMENS MAGNETOM with a 32-channel head coil (Siemens, Munich, Germany). A time-course series of 252 scans were acquired using T2*-weighted, gradient echo, echo-planar imaging (EPI) sequences. Each volume consisted of 96 slices, with a slice thickness of 1.5 mm with no gaps. The time interval between two successive acquisitions of the same image (TR) was 3,000 ms, the echo time (TE) was 40.2 ms, and the flip angle was 90°. The field of view (FOV) was 192 mm, and the matrix size was 128 × 128, giving voxel dimensions of 1.5 mm × 1.5 mm × 1.5 mm. Scan acquisition was synchronized to the onset of each trial. The total experimental duration was 756 s After functional scanning, structural scans were acquired using a T1-weighted gradient echo pulse sequence (TR = 1,900 ms; TE = 3.63 ms; flip angle = 8°; FOV = 180 mm; voxel dimensions of 0.6 mm × 0.6 mm × 0.6 mm) to extract the habenula regions. Whole-brain T1-weighted structural images were also acquired using a 3D magnetization-prepared rapid gradient echo (MP-RAGE) sequence (178 1-mm thick sagittal slices with a 256 × 256 acquisition matrix; the field of view, 256 mm; TI/TR/TE = 900/2,300/2.98 ms; flip angle, 9°; scan time, 5.12 min).

#### Analysis of Behavioral Data

We compared the proportion of low probability choices between background pictures (picture A and picture B) by conducting a paired *t*-test to examine whether background picture presentations would be associated with avoidance of painful stimuli. We analyzed the number of shape selection by using a binomial test to compare the proportion of low probability choices against the null hypothesis mean = 50%, both in background pictures. We also examined whether participants learned to choose the shape associated with avoidance of painful stimuli by conducting 2 (probability: high vs. low) × 2 (contextual information: picture A vs. picture B) ANOVAs on reaction times.

Furthermore, we examined correlations between the TCI scores and left and right habenula activation during aversive outcomes, based on the 7-factor psychobiological model of the TCI and asymmetry and lateralization of the habenula (Ahumada-Galleguillos et al., 2017; Ichijo et al., 2017). The Bonferroni correction indicated* p* < 0.05/7 = 0.0071.

#### Model-Based Analyses

To model participants’ learning processes, we adopted the Q-learning model, a standard reinforcement learning algorithm (Watkins and Dayan, 1992; Ito and Doya, 2009) as follows. For each participant, the action-value *Q*_t_ (*s, a*), which is the estimate of return obtained by taking an action *a* ∈ {*L, R*} (left or right shape) when an state *s* ∈ {*A, B*} (a background image A or B) is represented at the *t*-th trial, is updated by the following rule:

(1) $$Q_{t+1}(s+a)=Q_{t}(s,a)+\alpha RPE_{t}$$

$$RPE_{t}=\kappa R_{t}-Q_{t}(s,a),$$

where *R*_t_ ∈ {0, 1} is the dummy variable such that *R*_t_ = 1 (or *R*_t_ = 0) if the painful stimulus (or no painful stimulus) is given at the *t*-th trial. A parameter *a* is the learning rate (the step-size for the update), and a parameter *k* is the subjective magnitude of the painful stimulus (simply referred to as “subjective painfulness” later). *RPE*_t_ is an aversive prediction error signal representing the difference between actual and expected return (i.e., subjective painfulness in our context) incurred at the *t*-th trial.

Using the action values, the probability to choose the left shape (*a* = *L*) at the *t*-th trial is given by the following:

(2) $$P_{t}(a=L|s)=\frac{\exp[Q_{t}(s,a=L)]}{\exp[Q_{t}(s,a=L)]+\exp[Q_{t}(s,a=R)]}$$

Using Eqs. (1) and (2), we can define the state-value *V*_t_ (*s*), which is the expected return for a given state *s*, as follows:

(3) $$V_{t}(s)=\sum_{a\in \{L,R\}}P_{t}(a=L|s)Q_{t}(s,a)$$

Note the action- and state-values, as well as the action probability, vary depending on the choice of the model parameters (α, κ). To fit the model into individual behavior, the model parameters were determined by the maximum likelihood estimation such that

(4) $$(\alpha ,\kappa )=\begin{array}{c} \arg\max\\ \alpha \in D_{\alpha },\kappa \in D_{\kappa } \end{array}\sum_{t=1}^{T}\ln P_{t}(a_{t}|s_{t}),$$

where *a*_t_ and *s*_t_ are actual action and state at the *t*-th trial, and *T* is the total number of trials experienced by the participant. *D*_β_ and *D*_κ_ are the domains of parameters α and κ. Due to the numerical tractability and an assumption that the subjective painfulness corresponds to negative return, we sought for the optimal parameters on the grid of *D*_β_ ∈ {0.01, 0.02, …, 1.00} and *D*_κ_ ∈ {−10.0,…, −0.1, 0.0}. For the further analysis, the estimated state-value *V*_t_ (*s*) and the aversive prediction error *RPE*_t_ were used.

### fMRI Analysis

#### Definition of the Habenula and Hippocampal Regions of Interest (ROI)

##### Habenula

The left and right habenula in native space were created by manually tracing on the software MRIcron. We adopted the method described in detail by Lawson et al. (2013; Figure 2).

##### Hippocampus

Manual tracing for abstracting the left and right hippocampus in each participant was performed using the software MRIcron, guided by an anatomical atlas (Duvernoy, 2005), and the hippocampal anatomical ROI was drawn up. This tracing was mainly conducted based on a previous study (Loh et al., 2016) which had methods for hippocampal segmentation including manually segmenting the hippocampus on a generated group T1 template and for using Duvernoy’s anatomical atlas. We used the individual T1 structural images to match with the condition of habenula anatomical ROI.

##### Preprocessing and Event-Related Analysis

Functional MRI data were pre-processed using the SPM software package (SPM8, Wellcome Department of Cognitive Neurology, London). EPI data were slice-time corrected, motion-corrected, unwarped using a field-map of the static magnetic field, co-registered to their whole-brain anatomical scan and spatially smoothed with a 2 × 2 × 2 mm Gaussian kernel. The T1 structural images to enable localization of the habenula were also co-registered to their whole-brain anatomical scans (Figure 2).

Following data pre-processing, pre-processed BOLD data were analyzed using multiple regression, for each participant (first-level analysis). Task-related activity was identified by convolving a vector of the stimulus onset times with a synthetic hemodynamic response. The general linear model (GLM) was used to examine the effects of interest. We set the model specifications assigned to the following five conditions; the onset of the outcome “pain,” the onset of the outcome “no pain,” the onset of a shape on the background picture A, the onset of a shape on the background picture B, and the identity of the chosen shape, or “shape selection” at the time participants’ indicated their choice. Each item was modeled as stick functions. The aversive prediction errors were entered into the “pain” or “no pain” conditions, and state values were entered in the “shape selection” condition as parametric regressors. We conducted motion corrections by adding head realignment parameters to the model specification as regressors. Motion parameters for all six planes (x, y, z, roll, pitch, yaw) were examined, and all six motion parameters were entered as nuisance regressors. The maximum motion of all six metrics for each person ranged from 0.95 mm to 2.61 mm. The average motion across all participants was 0.3, 0.9, and 1.1 mm for x, y, and z, respectively. The average rotational movement throughout the task across all participants was 0.004, 0.005, and 0.005 degrees for roll, pitch, and yaw, respectively. To examine the differences in head motion during between painful stimulation and non-painful stimulation, we used aggregated values across all six metrics on the phase of the outcome “pain” or “no pain.” There were no differences in head motion during between painful stimulation and non-painful stimulation (*t* = 0.58, *p* = 0.56). A contrast image was created for each participant through a linear combination of the estimated beta images: the onset of the outcome “pain” minus the onset of the outcome “no pain.” We also examined the correlations between the BOLD signal of the habenula and aversive prediction errors or value scores by using a parametric modulation analysis (Büchel et al., 1998). For the habenula analysis, average contrast values (parameter estimate) were extracted from the left and right habenula ROIs, and one-sample *t*-tests were performed to identify activation of the left and right habenula using SPSS version 16.0. We also tested the association between habenula activation and clinical assessments (BDI-II, STAI, and TCI) using Pearson correlation coefficients.

**Figure 3:**
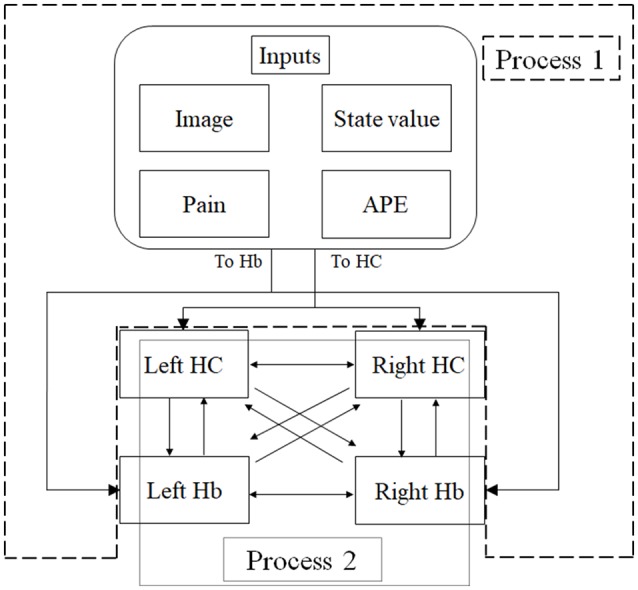
An overview of dynamic causal modeling (DCM). We tested multiple combinations of inputs, input regions, and connections by using this model, and chose the best combination that explains the BOLD signals using a Bayesian model selection procedure that computed the posterior probability (the probability of the model given the data) over the competing models that were used. First, we estimated inputs and input regions under the fully-connected brain regions (Process 1). Second, we estimated the effective connectivity between the brain regions under the estimated inputs and input regions (Process 2). APE, aversive prediction error; HC, hippocampus; Hb, habenula.

**Table 1:** The combination of inputs and target of inputs (“Process 1” on Figure 2).

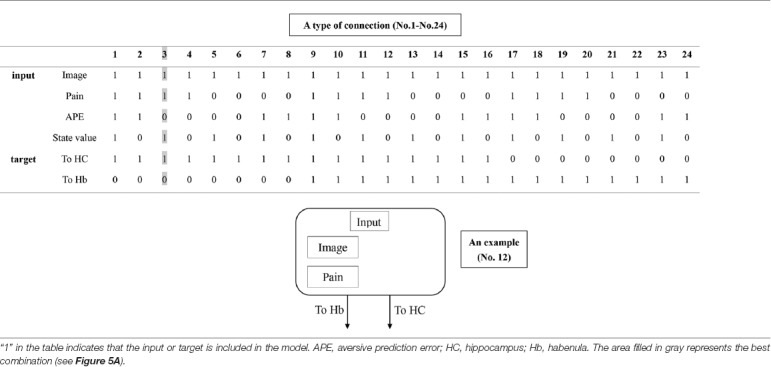

*“1” in the table indicates that the input or target is included in the model. APE, aversive prediction error; HC, hippocampus; Hb, habenula. The area filled in gray represents the best combination (see Figure 5A)*.

**Table 2:** The combination of connections (“Process 2” on Figure 2).

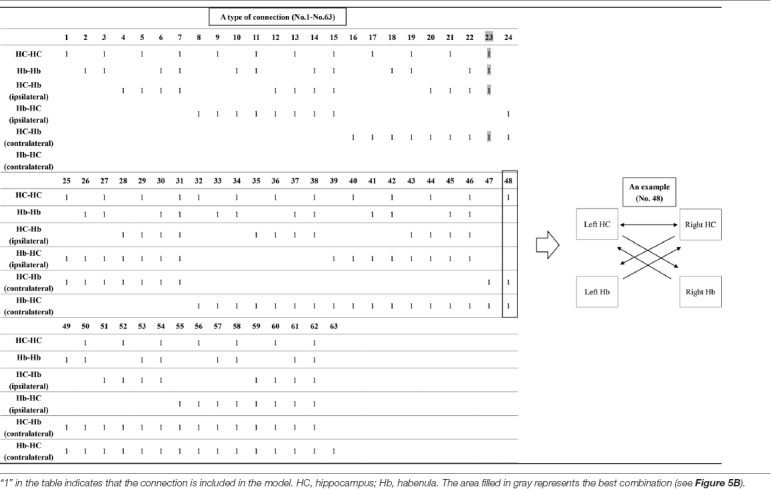

*“1” in the table indicates that the connection is included in the model. HC, hippocampus; Hb, habenula. The area filled in gray represents the best combination (see Figure 5B)*.

**Table 3:** Proportion of low probability choices and reaction time as functions of probability (high vs. low) and contextual information (picture A vs. picture B).

	Picture A		Picture B	
	High (M ± SD)	Low (M ± SD)	High (M ± SD)	Low (M ± SD)
Reaction time (ms)	1,237.6 ± 435.1	1,181.9 ± 387.8	1,210.4 ± 384.2	1,189.3 ± 366.0
Proportion of low probability choices (%)	61.2 ± 20.0		58.5 ± 20.6	

##### Dynamic Causal Modeling (DCM)

DCM is a procedure for identifying nonlinear systems that use Bayesian parameter estimates to make inferences about the effective connectivity between brain regions, as well as how this connectivity is affected by experimental conditions (Friston et al., 2003). Events’ inputs in DCM were modeled by the similar way we conducted for the habenula analysis of the univariate GLM. We used four regions (the left and right habenula and left and right hippocampus) and analyzed the effective connectivity between these brain regions using DCM. Previous studies have reported that the hippocampus has a critical, and a direct influence on contextual conditioning (Glenn et al., 2014; Davachi and DuBrow, 2015; Goujon et al., 2015) and that the habenula and hippocampus are closely connected during processing associated with contextual conditioning (Mizumori and Baker, 2017). However, no human studies in the relationship between the habenula and hippocampus have been conducted, and therefore, we examined the possibility of connectivity using DCM. We tested multiple combinations of inputs, input regions, and connections to estimate the effective connectivity between the brain regions (Figure 3) to select the best combination that explains the BOLD signals using a Bayesian model selection procedure that computed the posterior probability (the probability of the model given the data) over the competing models that were used (Friston et al., 2003; Stephan et al., 2009). Because of the large number of combinations, we divided the connectivity estimation process into two processes. First, we estimated inputs and input regions under the fully-connected brain regions (Process 1; Table 1 and Figure 3). Second, we estimated the effective connectivity between the brain regions under the estimated inputs and input regions (Process 2; Table 2 and Figure 3). We used the background images, pain, and state values of the image, as well as the aversive prediction errors as inputs. There were 24 combinations of these inputs and input regions (Table 1). After the best combination of inputs and input regions were estimated, we estimated the connectivity between the habenula and hippocampus (Table 2). Note that we assumed the connectivity between the regions was symmetric.

**Figure 4:**
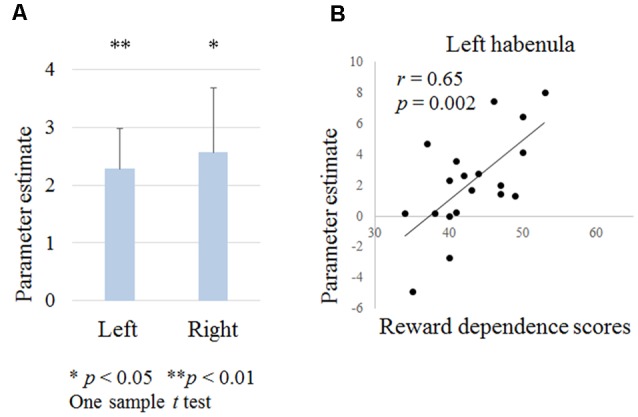
**(A)** Habenula activation during negative outcomes in contextual learning tasks. **(B)** Relationship between the left habenula activation during negative outcomes and temperament and character inventory (TCI) reward dependence scores.

## Results

### Behavioral Results

A paired *t*-test on the proportion of low probability choices between background pictures revealed no significant differences (Table 3) and both in background pictures, a higher proportion of low probability choices were shown (more than 50% of total choices; *p* < 0.001 by a binomial test).

There was no significant interaction effect or main effects of probability × contextual information on reaction times (Table 3).

### fMRI Data

#### Habenula Activation During Punishment Outcome

We examined the left and right habenula activation during punishment outcomes. A one-sample* t*-test revealed that the left and right habenula was activated (right habenula; *t* = 2.3, *p* = 0.033, left habenula; *t* = 3.2, *p* = 0.005; Figure 4A). There was no correlation between habenula activation and the age, the proportion of low probability shapes or the reaction time during shape selection.

#### Correlation Between the BOLD Signal of the Habenula or the Hippocampus and Aversive Prediction Errors

The left habenula was significantly correlated with the parametric regression coefficient for aversive prediction error (*t* = 2.1, *p* = 0.044). However, there was no significant relationship between the right habenula or the hippocampus and aversive prediction error. We also found no relationship between habenula activities and state values.

#### Relationship Between the Left and Right Habenula Activation and TCI Scores

Reward dependence scores were positively correlated with left habenula activation (*r* = 0.65, *p* = 0.002; Figure 4B). There were no correlations between other scores and habenula activation.

#### DCM Results

**Figure 5:**
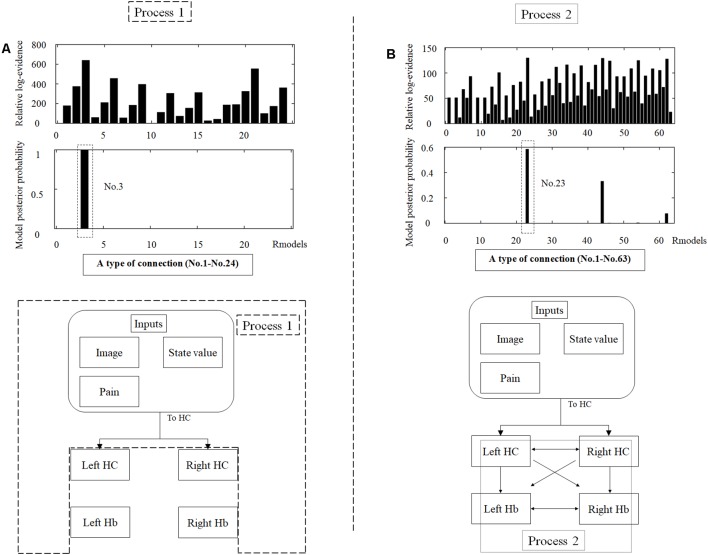
The tables in the upper part of the figure show the relative log-evidence and the posterior probability of models in the combination of inputs and input regions under the fully-connected brain regions (habenula and hippocampus; **A**) and the connectivity between the brain regions **(B)**. The horizontal axis in table A and table B show a type of connection defined in Tables s 1, 2, respectively. As inputs, we used background images, pain, and state values of the image, as well as the aversive prediction error (APE). As a result, the best model that had inputs of background images, pain, and state values to only the hippocampus (a type of connection; No. 3; Process 1), and that had connections from the hippocampus to habenula was selected among the competitive models (a type of connection; No. 23; Process 2).

**Figure 6:**
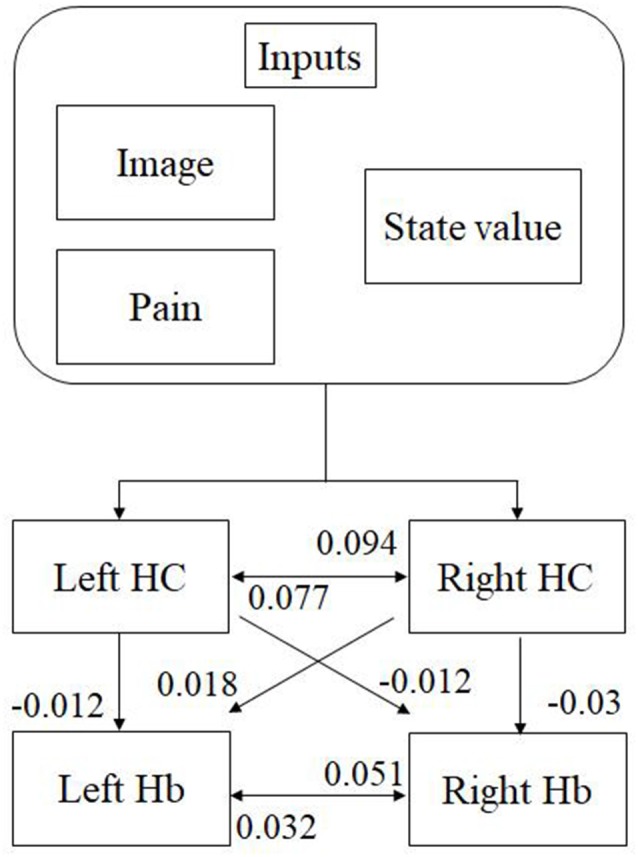
The most suitable model selected among the competitive models. The model has inputs of background images, pain, and state values only to the hippocampus, as well as connections from the hippocampus to habenula. The value shows intrinsic connectivity parameters.

We analyzed the effective connectivity between the habenula and hippocampus using DCM. First, we estimated inputs and input regions under the fully-connected brain regions. We compared the posterior probability of models with various combinations of inputs and inputs target regions (Table 1). As a result, the model with inputs of background images, pain, and state values only to the hippocampus was selected from the competitive models (Figures 5A, 6). We then estimated the connectivity between the four regions (the left and right habenula and left and right hippocampus) using inputs of background images, pain, and state values to the hippocampus that were selected in the previous procedure (Table 2). The results indicated that the model with connections from the hippocampus to habenula was the best model that explained the data in the competing models (Figures 5B, 6). These results indicated that the habenula and hippocampus are closely connected during the processing associated with contextual conditioning. Especially, as Figure 6 shows, there was a positive intrinsic connectivity parameter between the right hippocampus and left habenula.

## Discussion

This study was conducted to examine the neural mechanisms underlying human habenula functioning during contextual learning. We observed activation of the left and right habenula in response to negative outcomes or during aversive prediction errors, and the left and right hippocampal function closely correlated with the presentation of contextual information and the left and right habenula function. Furthermore, there were positive correlations between the left habenula activation during negative outcomes and TCI reward dependence scores. This is the first study to reveal habenula activation and functional connectivity between the habenula and hippocampus using a contextual learning task.

### Shape Selection Frequencies and Reaction Times for Behavioral Results

Behavioral results showed the increased choice of low punishment probability shapes and no changes in reaction times between low and high punishment probability shapes. An animal study has shown that modulation of dopamine type 1 receptors in lateral habenula neurons impaired contextual fear memory, particularly for the acquisition stage of the learning process (acquisition, consolidation, and retrieval; Chan et al., 2017). The acquisition is in the initial stage (trial and error) of the learning process. If the participants had already realized the aim of this task (e.g., a painful stimulus occurs more frequently if they select a rhombus for the background picture A), they would be able to conduct a suitable choice more quickly. Therefore, no reaction time changes and the increased choice of low punishment probability shapes in the present results would show that the learning process in this task was in the initial stage of trial and error, and we consider that it would be suitable for observing the activation of the habenula.

### Activation of the Habenula During Learning Processing

The results of the present study showed that the left and right habenula was significantly activated during negative outcomes in the form of aversive stimuli. In previous animal studies, it has been consistently reported that habenula activation is associated with learning-related processing, particularly when receiving negative reward signals (Matsumoto and Hikosaka, 2009; Hikosaka, 2010; Proulx et al., 2014; Hennigan et al., 2015). Furthermore, a rat study has shown that habenula functioning more strongly interacts with context-dependent learning than it does the processing of simple reward or punishment signals (Baker et al., 2015). There are various human fMRI studies of habenula functioning (Furman and Gotlib, 2016; Lawson et al., 2017; Liu et al., 2017), but as a problem, the tasks that are used in these studies involve mainly cue learning. Salas et al. have studied habenula activity to negative reward prediction errors in healthy human participants by setting the non-delivery of expected reward (juice; Salas et al., 2010). We consider that our study replicated *Salas et al’s* study that showed activation in human habenula to negative reward prediction errors. In addition to that, we also found that the habenula activation was associated with the hippocampus and an individual’s temperament. Our study identified the habenula morphologically as an extraction method and researched the functional connectivity with the hippocampus. We consider that it would be different from the Salas et al’s study that identified the habenula by computing correlation with striatal time series. Habenula fMRI studies are still lacking, although the habenula is believed to play an important role in various functions such as modulating reward, feeding behavior, drug withdrawal, pain, and sleep (Salas et al., 2009; Hikosaka, 2010). Therefore, we consider that it would be useful to study the habenula from a variety of perspectives, including our study. Contextual information influences performance during reversal learning tasks (Üngör and Lachnit, 2006), and in fact, a monkey study found that the habenula strongly activated in a reversal-learning task (Kawai et al., 2015). Therefore, we assumed to be able to extract the activation of the habenula in detail by adding such contextual information, and we consider that it is important to incorporate contextual information within a learning task to show activation of this brain region. In the present study, we identified left habenula activities that were consistent with aversive prediction errors, and therefore, we consider that the left habenula encoded those signals. Pathophysiological mechanisms of psychiatric disorders and chronic pain have been reported to be associated with habenula functioning (Hikosaka, 2010; Boulos et al., 2017), and we will conduct the same task used here with samples of these patients to examine such a mechanism.

### Relationship Between the Habenula and Hippocampus

The habenula links with many neural networks as a regional hub in animals including humans (Hikosaka, 2010). The functional connectivity between the habenula and hippocampus in vertebrates has been previously described (Loonen and Ivanova, 2016; Torrisi et al., 2017). Mizumori and Baker (2017) propose a brain network including the hippocampus and habenula as a core neural circuit that enables animals to perform adaptive behaviors during a learning task involving contextual information. Contextual conditioning that has been recognized as a spatial perception impacts behavioral choices in a learning task and the information has been reported to associate with the activation of the hippocampus (Glenn et al., 2014; Davachi and DuBrow, 2015; Goujon et al., 2015). This type of memory based on context has been associated with the hippocampal activity. For example, poor contextual memory of trauma is reported to result from disrupted hippocampal functioning (Glenn et al., 2014), and these mechanisms are believed to be linked to the etiology of posttraumatic stress disorder (Acheson et al., 2012). Therefore, we investigated the functional connectivity between the habenula and hippocampus in the present human learning task by using DCM, and we found such connectivity during contextual conditioning. Especially, we found a positive connectivity parameter between the right hippocampus and left habenula. Only the left habenula activity was correlated with aversive prediction errors and we speculate that it would be a possibility that the right hippocampus modulated the left habenula activity through contextual information. Based on this previous knowledge, it is possible that our results could shed light on Baker’s (2017) hypothesis in humans. We consider that these regions may be related with the above learning function. Furthermore, we found a significant relationship between aversive prediction errors and habenula activities, but not hippocampal activities. This result might provide a preliminary evidence for an interesting dissociation between the putative modulators of the hippocampus (i.e., image, pain, state value) and the prediction error signal represented in the left habenula.

### Relationship Between Habenula Activation and Reward Dependence Scores

Our results showed a positive relationship between the left habenula activation during punishment outcomes and TCI reward dependence scores. We hypothesized that higher activation of the habenula during the aversive learning process was related to low reward dependence, high novelty seeking, and high behavioral inhibition. However, the results did not support this hypothesis. Reward dependence is the rapid responsiveness to rewards, leading to maintaining ongoing behavior. Previous studies on rats about negative reward-related learning tasks were mainly related to novelty exploratory behavior (Shumake et al., 2003, 2005). On the other hand, this experiment examined the activation of the habenula during aversive outcomes, and the design could also be relevant to maintaining approach behavior because the participants had to select either one of the shapes for obtaining a reward (no pain) after accepting the outcome. Therefore, in the present study, button choices may be represented as maintaining approach behavior rather than novelty exploratory behavior, and our results may have been related to such differences in experimental design. The reason for the positive correlation between habenula activation during aversive outcomes and reward dependence scores could have occurred because people that have the temperament for making more approach behaviors to obtain a reward would tend to acquire more avoidance behaviors (i.e., the reward (no pain) in this experiment), resulting in the increased activation of the habenula.

### Limitation

The present study has several limitations. First, the small sample size used here suggests that our results might not be robust. Second, the hippocampus that we extracted is widespread, and elucidation of further local functionality regarding linkages with the habenula may be advisable. Third, it has been reported that the habenula has different functions in the medial and lateral regions especially in animal studies (Hikosaka, 2010). We were unable to research such a differentiation by our fMRI technique. Fourth, in the present study, we tested the DCM model, based on the previous studies (Glenn et al., 2014; Davachi and DuBrow, 2015; Goujon et al., 2015; Mizumori and Baker, 2017). However, we were unable to compare all possible models and so there may be an untested model that fits the data better. Finally, we did not examine differences in the behavioral results between our contextual learning task and a simple probabilistic reinforcement learning task. As a result, the additional effects of our task might have been unclear from a behavioral perspective, although we found an enhancement of habenula activation by the hippocampus, which is a region that is essential for contextual information. Further study is needed to advance the results found here, including investigations of potentially more effective techniques for imaging the human habenula and for probing learning processes dependent on habenula function and the differences between psychiatric disorder or chronic pain and healthy controls.

## Conclusion

We investigated how the habenula activates during a contextual learning task. We found activation during negative outcomes, the association with aversive prediction errors, a positive relationship between such habenula activation and TCI reward dependence scores, and functional connectivity between the habenula and hippocampus during the presentation of contextual content. We consider that the findings of habenula activity from animal studies of contextual learning would be replicated in humans and that the learning mechanism would involve linkages with the hippocampus. These brain regions may potentially play an important role in avoidance behavioral function.

## Data Availability Statement

The datasets generated for this study are available on request to the corresponding author.

## Ethics Statement

All participants gave their written informed consent before participation, according to the protocol reviewed and approved by the Ethics Committee of Hiroshima University. All procedures followed were under the ethical standards of the responsible committees on human experimentation (institutional and national) and with the Helsinki Declaration of 2013, and the appropriate revisions at the time of the investigation. Informed consent was obtained from all patients included in the study.

## Author Contributions

AY was involved in the conception and design of the study, acquisition and analysis of data, and drafting the manuscript or figures. MT, NI, YY, SYo, KK, TN, JY, and CS contributed to the acquisition and analysis of data. YS, GO, YO, HA, and SYa contributed to the design of the study and revision of the manuscript.

## Conflict of Interest

AY previously received support in other research from Eli Lily. The remaining authors declare that the research was conducted in the absence of any commercial or financial relationships that could be construed as a potential conflict of interest.
